# *Bacillus anthracis* Bioterrorism Incident, Kameido, Tokyo, 1993

**DOI:** 10.3201/eid1001.030238

**Published:** 2004-01

**Authors:** Hiroshi Takahashi, Paul Keim, Arnold F. Kaufmann, Christine Keys, Kimothy L. Smith, Kiyosu Taniguchi, Sakae Inouye, Takeshi Kurata

**Affiliations:** * National Institute of Infectious Diseases, Tokyo, Japan; † Northern Arizona University, Flagstaff, Arizona, USA; ‡ Stone Mountain, Georgia, USA

**Keywords:** Bacillus anthracis, bioterrorism, anthrax, epidemiology, Aum Shinrikyo, Japan

## Abstract

In July 1993, a liquid suspension of *Bacillus anthracis* was aerosolized from the roof of an eight-story building in Kameido, Tokyo, Japan, by the religious group Aum Shinrikyo. During 1999 to 2001, microbiologic tests were conducted on a liquid environmental sample originally collected during the 1993 incident. Nonencapsulated isolates of *B. anthracis* were cultured from the liquid. Multiple-locus, variable-number tandem repeat analysis found all isolates to be identical to a strain used in Japan to vaccinate animals against anthrax, which was consistent with the Aum Shinrikyo members’ testimony about the strain source. In 1999, a retrospective case-detection survey was conducted to identify potential human anthrax cases associated with the incident, but none were found. The use of an attenuated *B. anthracis* strain, low spore concentrations, ineffective dispersal, a clogged spray device, and inactivation of the spores by sunlight are all likely contributing factors to the lack of human cases.

## Incident

On June 29, 1993, five residents of Kameido, Koto-ward, an eastern area of Tokyo, reported foul odors to local environmental health authorities. On investigation, officials found that the odors originated from the eight-story headquarters building of the religious group Aum Shinrikyo. The group was suspected of abducting several escaped members and anti–Aum Shinrikyo activists; however, lacking strong evidence of criminal activity, national security and law enforcement authorities had not restricted Aum Shinrikyo’s activities.

On June 30, the local environmental health authority registered 41 complaints that foul odors were causing appetite loss, nausea, and vomiting in some exposed persons. Because of the complaints, officials requested permission to inspect the building’s interior, but Aum Shinrikyo members refused. Officials checked the building’s surroundings, collected air samples, and began to survey activity at the building, but other than the nuisance posed by the odor, no readily apparent risk to human health could be found.

**Figure 1:**
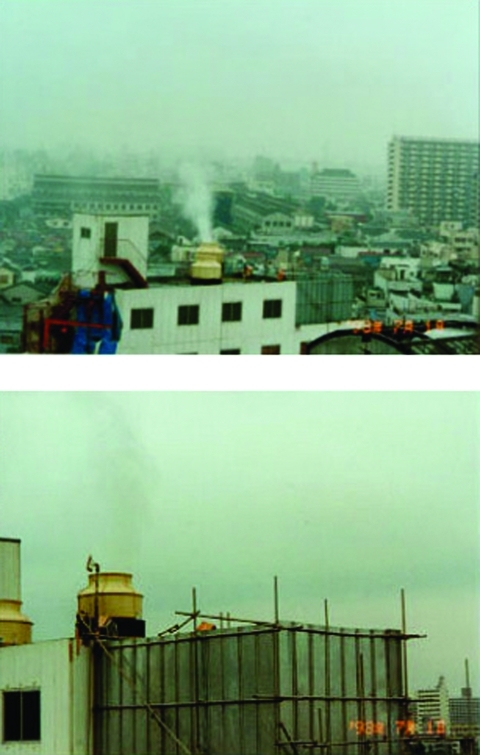
Spraying scenes from the Aum Shinrikyo headquarters building (photographs taken July 1, 1993, by the Department of Environment, Koto-ward).

On the morning of July 1, neighbors reported loud noises and an intermittent mist emanating from one of two cooling towers on the building’s roof (Figure 1). As the day progressed, residents (mostly living south of the building) lodged 118 complaints about the foul odors with the environmental health office. Light rain fell early in the day (a total of 7 mm, 1 mm each hour from 1:00–7:00 a.m.). Wind (2–4 m/sec) blew from north-northeast to northeast in the morning and northeast to east-northeast in the afternoon. The minimum and maximum temperatures were 16.9°C at 3:00 a.m. and 4:00 a.m. and 19.9°C at 3:00 p.m., respectively. The day was rainy and cloudy, with no direct sunlight.

The same day, residents in the neighborhood reported a “gelatin-like, oily, gray-to-black” fluid from the mist from the cooling towers collecting on the side of the building. Environmental officials collected samples of this fluid and stored them in a refrigerator (4°C) for later testing.

Intermittent misting continued until demands from local residents forced Shoko Asahara, founder of Aum Shinrikyo, to agree on the morning of July 2 to cease using the rooftop device and to clean and vacate the building. No equipment remained when officials inspected the building on July 16, although they noted black stains on the walls.

This incident was largely forgotten until, in the aftermath of the March 1995 sarin gas attack on the Tokyo subway system, police investigations uncovered evidence that Aum Shinrikyo was involved in bioterrorism. The true nature of the Kameido incident was not revealed to the public until Asahara was arraigned on May 23, 1996. Aum Shinrikyo members testified that the odors were caused by their efforts to aerosolize a liquid suspension of *Bacillus anthracis* in an attempt to cause an inhalational anthrax epidemic. They believed this epidemic would trigger a world war and lead to Asahara’s ruling the world.

At the time of the incident, the illnesses reportedly associated with the release were not well studied. In particular, no one sought evidence of inhalational anthrax or other syndromes caused by the anthrax bacillus, since the true nature of the mist was not recognized. Reports of short-term loss of appetite, nausea, and vomiting (symptoms not typical of *B. anthracis* infection) among some residents were the only contemporary evidence of human illness associated with the incident. Vague reports of illness in birds and pets were also noted in local media (*1*), but the exact nature of these illnesses remained unclear.

## Laboratory Findings

In November 1999, after long negotiation, local environmental authorities agreed that the one remaining fluid sample, collected as part of the 1993 investigation, could be tested for microbiologic pathogens. The test tube, which contained 2.6 mL of a turbid, gray-to-black fluid, was transferred to Northern Arizona University in Flagstaff, Arizona, for testing to identify and characterize its microbial flora.

**Figure 2:**
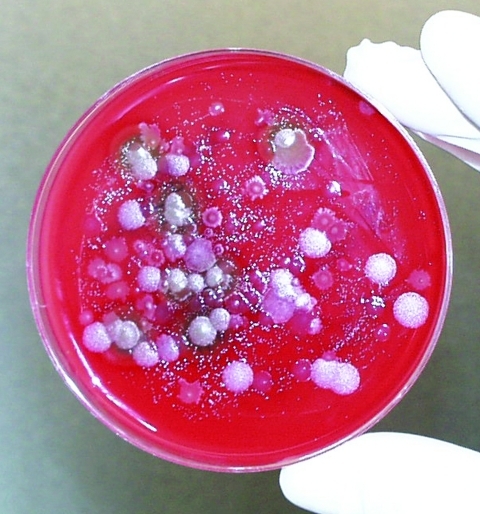
Fluid collected from the Kameido site cultured on Petri dishes to identify potential *Bacillus anthracis* isolates.

Provisional microscopy examination of the fluid stained by malachite green/safranin showed bacterial spores, a large amount of debris, and vegetative bacterial cells. Aliquots of the fluid were streaked on sheep blood agar plates and incubated aerobically at 37°C. After overnight growth, the plates were found to contain mixed bacterial flora; approximately 10% of the colonies were similar in appearance to *B. anthracis*. Suspect colonies were nonhemolytic and had the “gray ground glass” appearance typical of *B. anthracis* (Figure 2). Based on the number of colonies on the plates, the original liquid suspension had about 4 x 10^3^ colony-forming units (CFU) of the *B. anthracis*–like agent per milliliter.

A representative selection of 48 colonies of the *B. anthracis*–like agent was purified by single colony streaking and then subjected to multiple-locus, variable-number tandem repeat analysis (MLVA). The MLVA polymerase chain reaction (PCR) primers are specific for eight amplicon sites unique to *B. anthracis* (*2*), two of which are plasmids carrying genes for anthrax toxin (pX01) and capsule (pX02). Amplicon-size patterns are diagnostic for particular diversity groups and strains within *B. anthracis* (*3*).

**Figure 3:**
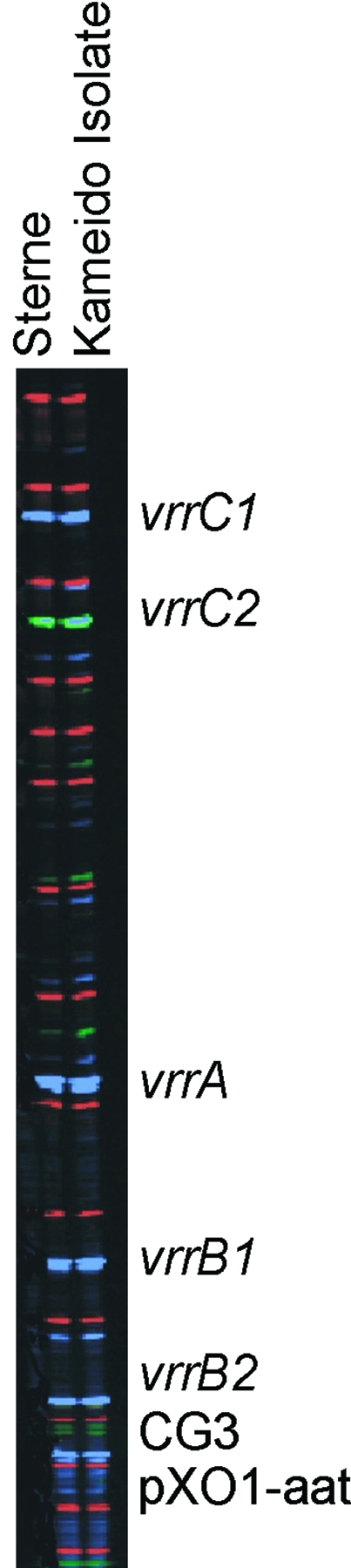
Multiple-locus, variable-number tandem repeat analysis genotype of all 48 Kameido isolates and the Sterne strain of *Bacillus anthracis*: *vrrA*, 313 bp; *vrrB*_1_, 229 bp; *vrrB*_2_, 162 bp; *vrrC*_1_, 583 bp; *vrrC*_2_, 532 bp; CG3, 158, bp; pX01-att, 129 bp; pX02, no amplification.

Analysis of the 48 suspect colonies confirmed them to be *B. anthracis*, and all had the same genotype. Of the eight marker sites, one—the locus for the pX02 plasmid coding for the anthrax capsule—consistently failed to amplify (*3*). All colonies contained the pX01 plasmid (coding for anthrax toxin) but lacked the pX02 plasmid (Figure 3). This genotype was identical to that of the Sterne 34F2 strain, used commercially in Japan to vaccinate animals against anthrax.

## Epidemiologic Findings

In Japan, culture-confirmed human anthrax is on the national notifiable disease list, and physicians are required to report all cases to the government. During the 1990s, only four human anthrax cases were reported (*4*). One of these cases, in Tokyo in August 1994, was in a man in his eighties from Sumida-ward, adjacent to Koto-ward (the location of Kameido); however, this case had no apparent association with the July 1993 incident.

**Figure 4:**
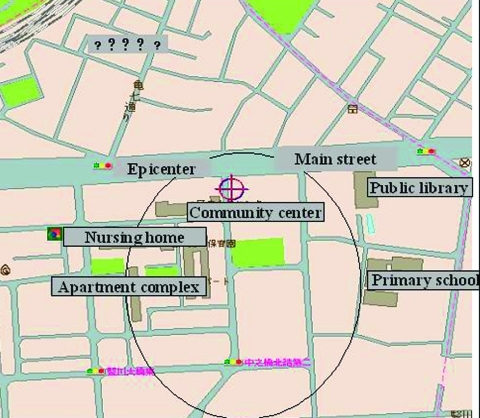
High-risk area for infection, based on foul odor complaints.

A retrospective case-detection survey was conducted to assess the possibility that some anthrax cases might have gone unrecognized or unreported. Using the official “foul odor” complaints as a guide, residences of the 118 complainants from July 1, 1993, were mapped to identify the area with the presumed highest risk for infection (Figure 4). The high-risk area included a 7-digit zip code area (0.33 km^2^) in Kameido, containing approximately 3,400 households and 7,000 residents. In 1999, physicians at 39 medical facilities (15 internal medicine, 7 dermatology, and 17 other specialties) serving the high-risk area were surveyed by telephone. None of these physicians reported having seen cases of anthrax, unexplained serious respiratory illnesses, or hemorrhagic meningitis, which is often a complication of systemic anthrax (*5*,*6*) in residents of the high-risk area.

## Discussion

The Kameido incident is the first documented instance of bioterrorism with an aerosol containing *B. anthracis*. Aum Shinrikyo members testified in 1995 that they were working with *B. anthracis*, but 6 years passed before the strain was isolated and characterized (*3*).

Why the Kameido incident failed to produce any documented cases of anthrax has not been fully explained, but the basis may be multifactorial. A virulent strain of *B. anthracis*, a sufficient concentration to cause disease, effective aerosolization, and favorable weather conditions would all have been necessary to produce the anthrax epidemic Aum Shinrikyo members said they wanted.

Molecular subtype analysis results demonstrate that the strain used in Kameido was a vaccine strain of *B. anthracis* without the ability to produce a protective capsule (*3*). This strain is generally regarded as nonpathogenic for immunocompetent people and is widely used in livestock without adverse consequences. Even if the strain had been virulent, however, the concentration of spores in the liquid suspension (10^4^/mL) was significantly less than the 10^9^ to 10^10^ organisms/mL considered to be optimal in a liquid-based biologic weapon.

The viscosity of the suspension was also greater than desirable. Successfully weaponizing anthrax spores requires creating a fine-particle cloud with a sufficiently high concentration of *B. anthracis*. The human respiratory infectious dose 50 (dose that will produce an infection in 50% of exposed persons) is unknown but has been estimated to be 8,000 to 10,000 spore-bearing particles <5 μm in diameter (*7*). Kameido residents described a gelatinous substance, suggesting the suspension would be poorly dispersed and droplets would be too large to form particles <5 μm in diameter. Additionally, the effectiveness of the spray system (code named “Water Mach” by the Aum Shinrikyo) was questionable; it apparently broke down repeatedly, and hydrostatic back pressure caused the suspension to leak from tubing used to transport it up eight stories. The spray head may have clogged with the high-viscosity fluid, contributing to back pressure.

Climate could have been another mitigating factor. While *B. anthracis* spores resist many environmental influences, they are killed by sunlight (*7*), with an estimated survival time in direct sunlight in July of <2.5 h. In Kameido, survival time may have been longer since the weather was overcast on July 1 (the day the mist was reported), but spore inactivation by solar radiation would still have reduced the already-low potential for infection.

Because of the associated foul odor, residents quickly detected a problem, but local officials did not suspect Aum Shinrikyo of developing biologic weapons, and they conducted no microbiologic examination at the time. The actual cause of the foul odor remains undetermined, but it may have been caused by heating the medium used to grow *B. anthracis* or failing to wash the medium from the suspension before dispersal. Geographic distribution of complaints about the odor corresponded with expected dispersal patterns of the aerosol under prevailing weather conditions. Infection risk could theoretically extend beyond the area of the foul odor complaints; however, focusing the telephone survey on this “high-risk” area provided the greatest chance of finding related cases of anthrax. Routine disease reporting by Tokyo-area medical association members did not provide evidence of potentially related cases outside the high-risk area.

## Conclusions

The Kameido investigation first showed the value of a high-resolution subtyping system for *B. anthracis* in forensic investigations. Its value was confirmed during investigations of the “anthrax letters” mailed to several persons in the United States in 2001 (*6*).

The details of Aum Shinrikyo activities led to a wider appreciation that subnational organizations may use biologic agents as weapons. Awareness is especially important in being prepared for a bioterrorist attack, since recognizing its nature early can substantially reduce associated sickness and death (*8*,*9*). Early recognition, however, requires training health professionals to recognize these diseases, having laboratories available to rapidly confirm clinical suspicions, and developing an active national surveillance program. Countries must also be able to rapidly deploy trained medical personnel, medical materials, and epidemiologists to affected communities. Most countries will need coordination among government agencies and private facilities with expertise relevant to the agents involved. To be effective, these measures require ongoing planning, preparation, and practice.
