# Correction, Vol. 10, No. 1

**DOI:** 10.3201/eid1002.C11002

**Published:** 2004-02

In the article “*Bacillus anthracis* Incident, Kameido, Tokyo, 1993” by Hiroshi Takahashi, et al., errors occurred in the 4th paragraph under “Discussion” on page 119: mu symbols were inadvertently replaced by the letter “m.” The corrected sentences appear below:

The human respiratory infectious dose 50 (dose that will produce an infection in 50% of exposed persons) is unknown but has been estimated to be 8,000 to 10,000 spore-bearing particles <5 μm in diameter (*7*). Kameido residents described a gelatinous substance, suggesting the suspension would be poorly dispersed and droplets would be too large to form particles <5 μm in diameter.

In addition, the name of the lead author of this article is misspelled in the table of contents of this issue. In the table of contents, the article should be attributed to “H. Takahashi et al.”

The corrected article appears online at http://www.cdc.gov/ncidod/EID/vol10no1/03-0238.htm

We regret any confusion these errors may have caused.

